# Case Report: Subcutaneous sensor-augmented insulin pump therapy in a 510-g preterm infant with hyperglycemia

**DOI:** 10.3389/fped.2026.1858894

**Published:** 2026-06-18

**Authors:** Christian Schlunk, Patrick Neuberger, Christina Künle, Martin Holder, Neysan Rafat

**Affiliations:** 1 Department of Neonatology, Olgahospital, Klinikum Stuttgart, Stuttgart, Germany; 2 Department of Paediatric Endocrinology and Diabetology, Olgahospital, Klinikum Stuttgart, Stuttgart, Germany

**Keywords:** continuous glucose monitoring, continuous subcutaneous insulin infusion, extremely low birth weight, neonatal hyperglycemia, off-label use, preterm infant

## Abstract

Neonatal hyperglycemia affects up to 80% of extremely low birth weight (ELBW) infants and is independently associated with mortality, intraventricular hemorrhage, and adverse neurodevelopmental outcomes. Intravenous insulin infusion, which is the standard of care, requires dedicated vascular access. To the best of our knowledge, this is the first report of combined continuous glucose monitoring (CGM) and continuous subcutaneous insulin infusion (CSII) in an ELBW infant (birth weight <1,000 g). Transient hyperglycemia developed on day of life (DOL) 2, necessitating insulin therapy. A 55-min intravenous bridge facilitated the transition to a subcutaneous sensor-augmented pump system (Medtronic MiniMed 780G; Guardian 4 CGM sensor; insulin aspart, undiluted). Given a subcutaneous adipose tissue depth of approximately 1–2 mm, standard insertion devices were not applicable; tangential near-horizontal manual insertion by a physician achieved reliable subcutaneous positioning. Over 14 days, 3,931 CGM measurements were recorded; the mean glucose level was 162 ± 57 mg/dL (9.0 ± 3.2 mmol/L), and the time in the target range (62–180 mg/dL; 3.4–10.0 mmol/L) was 59.4%. CGM reduced the number of invasive reference glucose measurements from an estimated 84–168 to 20, representing a 76%–88% reduction in cutaneous punctures. Infusion set occlusion on DOL 6 was identified prospectively using CGM trend analysis. Combined CGM and CSII were maintained uninterrupted throughout laparotomy for focal intestinal perforation, allowing real-time perioperative glycemic surveillance and proactive insulin dose titration without additional invasive procedures. Insulin therapy was discontinued on DOL 17 as glucose levels normalized. This case establishes proof-of-concept for sensor-augmented subcutaneous insulin pump therapy at the physiological extreme of viability, providing a procedural foundation for neonatal-adapted closed-loop trials.

## Introduction

Neonatal hyperglycemia—defined as blood glucose >150 mg/dL (8.3 mmol/L) (1)—affects up to 80% of extremely low birth weight (ELBW) infants (≤1,000 g), driven by β-cell immaturity, peripheral insulin resistance, and iatrogenic glucose load. Sustained hyperglycemia independently predicts mortality, intraventricular hemorrhage, and adverse neurodevelopmental outcomes (2, 3).

Intravenous continuous insulin infusion (IVII) remains the standard of care (4, 5) and requires dedicated vascular access and delivers only 56% of the prescribed dose within 2 h due to adsorption onto syringe barrels and PVC tubing (6–8). In addition, 12-hourly syringe changes with repeated insulin dilution are required. Subcutaneous pump delivery circumvents these limitations, with infusion sets certified for up to 7 days according to the manufacturer’s specifications. Continuous glucose monitoring (CGM) and continuous subcutaneous insulin infusion (CSII) have individually demonstrated feasibility in ELBW infants (9–12). However, their combined use as sensor-augmented pump therapy has not been reported in infants with a birth weight below 1,000 g.

## Case presentation

### Maternal history and antenatal course

A 39-year-old primigravida was referred at 24 weeks of gestation for a monochorionic–diamniotic twin pregnancy complicated by feto-fetal transfusion syndrome and twin anemia–polycythemia sequence (TAPS). Fetoscopic laser photocoagulation at 20 weeks (Quintero stage II) resulted in donor/recipient reversal. The former donor (Twin 1) developed intrauterine growth restriction, anemia, and cerebral injury, prompting a multidisciplinary consensus for palliative care. Antenatal corticosteroids were administered according to the national guidelines. Preterm premature rupture of membranes occurred 4 days before delivery, and incipient maternal pre-eclampsia precipitated an urgent cesarean section at 24 + 3 weeks.

### Delivery and immediate postnatal course

Twin 2—the subject of this report—was born female, weighing 510 g (8th percentile), with a crown-heel length of 32.0 cm and a head circumference of 21.0 cm. The Apgar scores were 7, 8, and 9 at 1, 5, and 10 min, respectively, and the umbilical artery pH was 7.35. Resuscitation included non-invasive respiratory support, poractant alfa via less-invasive surfactant administration, and inhaled nitric oxide (iNO) for persistent pulmonary hypertension. The first postnatal blood gas analysis showed a pH  of 7.20, pCO₂ of 64 mmHg, hemoglobin of 22.0 g/dL, hematocrit of 67%, and lactate of 3.6 mmol/L, consistent with polycythemia in a former blood transfusion acceptor. The initial blood glucose level was 42 mg/dL (2.3 mmol/L).

### Neonatal course

Non-invasive ventilation was maintained until DOL 10, when respiratory deterioration required intubation, and extubation was achieved on DOL 30 following dexamethasone treatment. Myocardial hypertrophy (TAPS-associated polycythemia and corticosteroids) was managed with propranolol. *Candida lusitaniae* septicemia (DOL 8; treated with fluconazole and micafungin), focal intestinal perforation (DOL 11; managed with laparotomy and divided stoma), and catheter-associated *Staphylococcus capitis* bacteremia (DOL 16) occurred. At the time of reporting, the infant was receiving high-flow nasal cannula therapy without supplemental oxygen. An intravitreal anti–vascular endothelial growth factor (anti-VEGF) injection was performed on the right eye for retinopathy of prematurity stage 3 pre-plus disease, and stoma closure was planned. The neurological examination is age-appropriate without focal deficits.

### Diagnosis and initiation of insulin therapy

Hyperglycemia exceeding 180 mg/dL (10.0 mmol/L) was first documented on DOL 2. Reducing parenteral glucose to 5 g/kg/day did not normalize blood glucose levels. Continuous insulin therapy was initiated with a 55-min intravenous bridge (Huminsulin Normal, 100 IU/mL; 0.1 IU/kg/h), during which subcutaneous device placement was performed, and written parental consent for off-label use was obtained.

### Device selection, preparation, and subcutaneous placement

**Figure 1:**
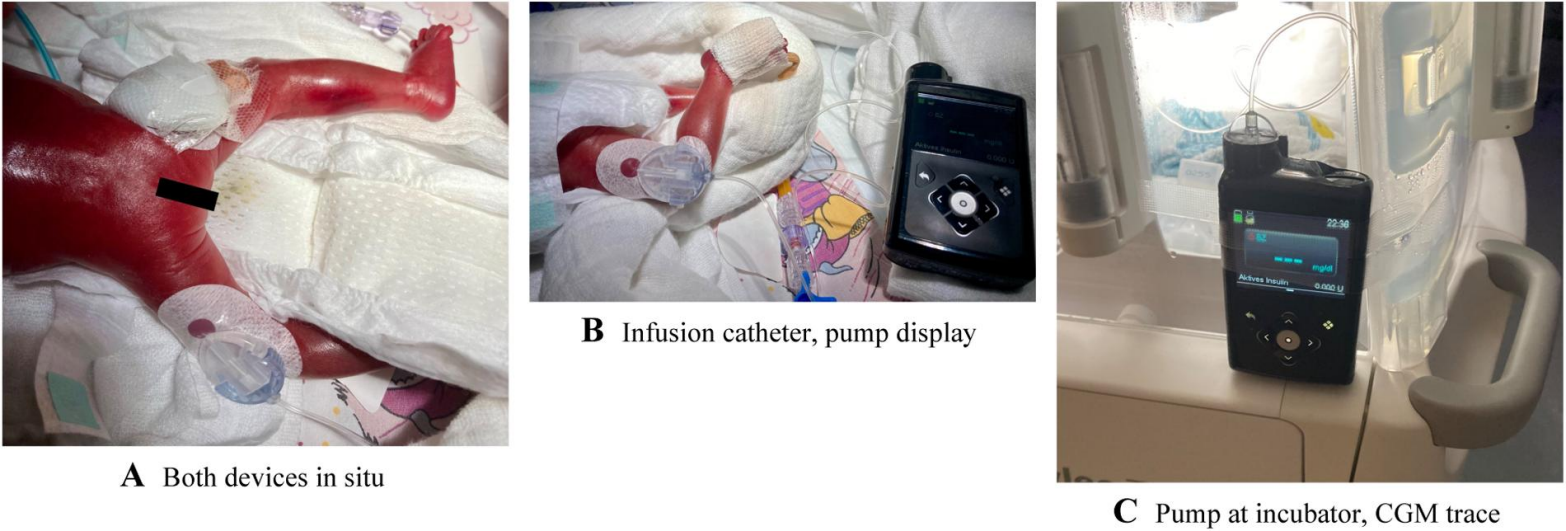
Subcutaneous device placement in an extremely low-birth-weight infant (510 g, 24 + 3 weeks of gestation). **(A)** Simultaneous *in situ* CGM sensor (left anterolateral thigh, Guardian 4) and subcutaneous infusion catheter (right anterolateral thigh, MiniMed Mio 30). **(B)** Subcutaneous infusion catheter (right anterolateral thigh, MiniMed Mio 30). **(C)** MiniMed 780G insulin pump positioned on the incubator wall. Images were anonymized, and written parental consent for photographic documentation and publication was obtained.

Subcutaneous insulin infusion was commenced using a MiniMed 780G pump (Medtronic, Northridge, CA) in manual mode. Insulin aspart (NovoRapid, 100 IU/mL; undiluted) was used as the infusate. The infusion catheter (MiniMed Mio 30, 6-mm soft cannula; Medtronic) was placed on the right anterolateral thigh. A Guardian 4 CGM sensor with a transmitter (Medtronic) was placed on the contralateral anterolateral thigh. Given the subcutaneous adipose tissue depth of approximately 1–2 mm, standard spring-loaded insertion was not applicable, and tangential near-horizontal manual insertion by a physician achieved reliable subcutaneous positioning (Figure 1). Two preconfigured basal rates (0.025 and 0.05 IU/h) simplified bedside operation; glucose alarms were set at 60 and 250 mg/dL (3.3 and 13.9 mmol/L). Dose adjustments were guided by CGM trends and verified by capillary glucometry (Accu-Chek Performa) and photometric analysis (HemoCue Glucose 201 DM).

### Glycemic course and CGM-guided management

**Figure 2:**
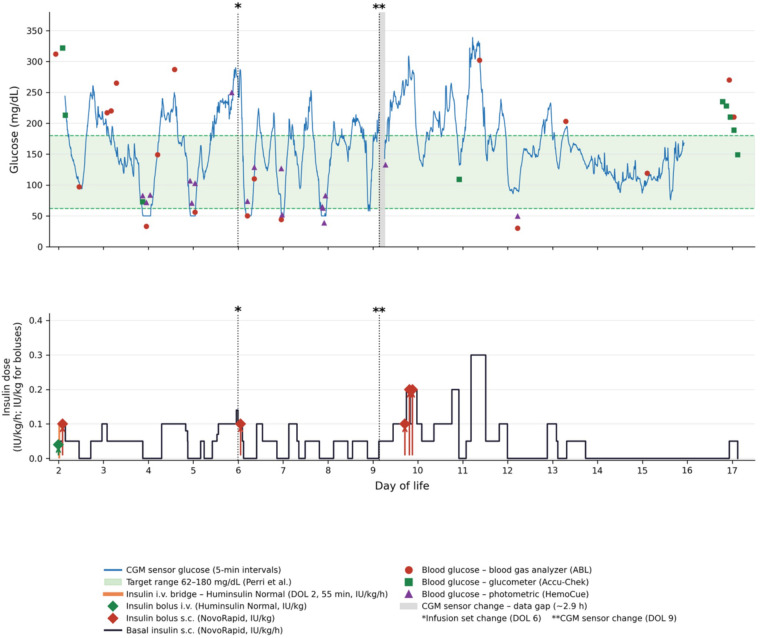
Glucose and insulin profiles during sensor-augmented pump therapy (day of life 2–17). Upper panel: Continuous glucose monitoring trace (blue line, 5-min intervals) with manual reference glucose measurements (blood gas analyzer, circles; glucometer, squares; photometric analyzer, triangles). The shaded green area indicates the target glucose range of 62–180 mg/dL (3.4–10.0 mmol/L). The gray zone represents the CGM data gap during the sensor change (day of life 9; duration 2.9 h). Lower panel: insulin dose (IU/kg/h) as a step function. The orange horizontal bar indicates intravenous regular human insulin bridge therapy. The arrows indicate the subcutaneous insulin boluses. *Infusion set change due to occlusion (day of life 6). **CGM sensor change (day of life 9).

**Figure 3:**
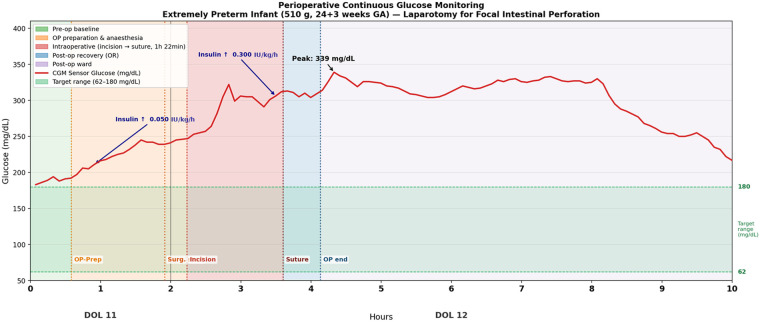
Perioperative continuous glucose monitoring trace and subcutaneous insulin basal rate during laparotomy for focal intestinal perforation (DOL 11–DOL 12; 10.5-h window). CGM sensor glucose (blue line, 5-min intervals); green shading = target range (62–180 mg/dL; 3.4–10.0 mmol/L). The vertical dashed lines indicate the operative phases (pre-operative preparation, incision, suture, and end of surgical aftercare). Subcutaneous insulin basal rate (IU/kg/h); purple arrows indicate dose escalation steps; 126 CGM readings were obtained across the observation window. DOL, day of life.

**Figure 4:**
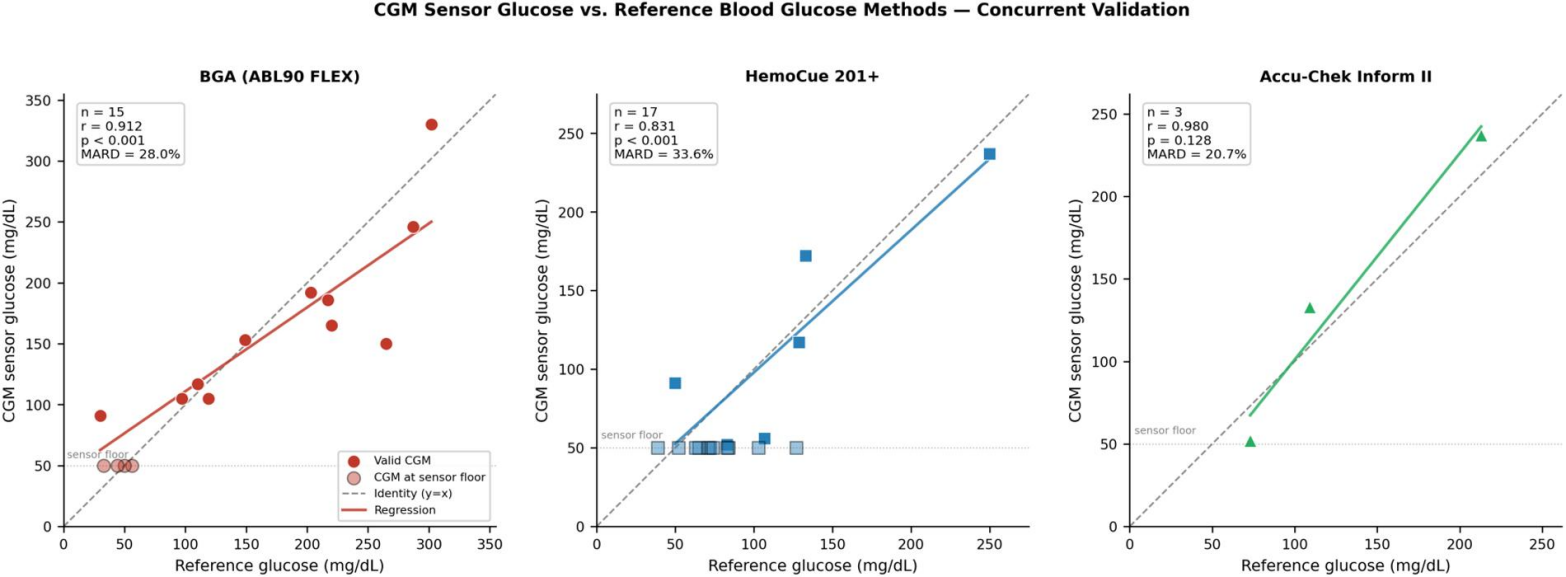
Concurrent validation of the CGM sensor glucose against three reference blood glucose measurement methods. Scatter plots show CGM sensor glucose (*y*-axis) versus reference glucose (*x*-axis) for the blood gas analyzer (ABL800 FLEX; *n* = 15, red circles), HemoCue Glucose 201 DM Analyser (*n* = 17, blue squares), and Accu-Chek (*n* = 3, green triangles). The dashed diagonal represents the identity line (y = x), and the solid lines show the linear regression calculated from the readings above the sensor floor. Faded markers indicate CGM values at the sensor detection floor (≤50 mg/dL), which were excluded from the regression analysis. The statistical parameters are annotated per panel. MARD, mean absolute relative difference; r, Pearson’s correlation coefficient.

Over 14 days (DOL 2–16), 3,931 CGM measurements were obtained at 5-min intervals (Figure 2). CGM metrics are presented in Table 1. On DOL 6, a glucose increase from 237 to 290 mg/dL (13.2–16.1 mmol/L) despite escalating doses—a pattern inconsistent with pharmacological resistance—indicated infusion set occlusion, which was confirmed by catheter replacement. During laparotomy (DOL 11–12), CGM and CSII remained *in situ* uninterrupted throughout all operative phases (Figure 3); the CGM sensor was replaced on DOL 9 (2.9-h data gap). Over the full monitoring period, 4 cutaneous punctures and 20 reference measurements were required, versus an estimated 84–168 under conventional 2–4-hourly monitoring (reduction 76%–88%). The lowest reference glucose level was 39 mg/dL (2.2 mmol/L) on DOL 7, without clinical signs. Insulin was discontinued on DOL 17 as glucose levels normalized. Concurrent CGM validation against three reference methods demonstrated clinically acceptable agreement (Figure 4): against blood gas analyzer (ABL800 FLEX; *n* = 15) *r* = 0.912 [mean absolute relative difference (MARD) 28.0%]; against HemoCue Glucose 201 DM (*n* = 17) *r* = 0.831 (MARD 33.6%); and against Accu-Chek Performa (*n* = 3) *r* = 0.980 (MARD 20.7%). The Guardian 4 sensor detection floor of 50 mg/dL (as per manufacturer’s specifications) accounted for a disproportionate share of the observed MARD. Above this detection floor, systematic bias was modest, reaffirming the need for confirmatory reference measurements in hypoglycemia management. Statistical analyses were conducted using Python 3 (scipy.stats); MARD = |CGM−ref|/ref × 100%. Floor events (≤50 mg/dL) were excluded from the regression.

**Table 1:** CGM-derived glycemic metrics over 14 days of sensor-augmented insulin pump therapy (DOL 2–16).

Metric	Value
No. of CGM measurements	3,931
Mean glucose (mg/dL) (mmol/L)	162 ± 57 (9.0 ± 3.2)
TIR (62–180 mg/dL, 3.4–10.0 mmol/L, %)	59.4
TAR (>180 mg/dL, >10.0 mmol/L, %)	35.6
TBR (<62 mg/dL, <3.4 mmol/L, %)	5.0
Minimum sensor glucose (mg/dL) (mmol/L)	50 (2.8)
Maximum sensor glucose (mg/dL) (mmol/L)	339 (18.8)
Total cutaneous punctures (device insertion + blood glucose measurements)	24

All metrics were derived exclusively from the CGM sensor glucose data (*n* = 3,931 measurements). CGM, continuous glucose monitoring; TIR, time in range; TAR, time above range; TBR, time below range.

## Discussion

To our knowledge, this is the first reported application of combined CGM and CSII in an ELBW infant weighing below 1,000 g, the smallest patient in whom sensor-augmented pump therapy has been deployed.

Several case series have previously described CSII alone in ELBW neonates with birth weights as low as 415 g and gestational ages as early as 23 + 5 weeks (12–14), but none used CGM concurrently. Perri et al. demonstrated CGM accuracy in very low birth weight (VLBW) neonates on parenteral nutrition and established reference glycemic percentiles. The lower target limit of 62 mg/dL (3.4 mmol/L) corresponds to the 10th percentile from this study, while the upper limit of 180 mg/dL (10.0 mmol/L) reflects our institution's threshold for insulin therapy initiation (9).

Insulin aspart was selected for its pharmacokinetic advantages: subcutaneous onset is 10–15 min (peak 40–50 min) versus 30–60 min (peak 80–120 min) for regular human insulin (as per the Summary of Product Characteristics, NovoRapid; Novo Nordisk). The high density of CGM data, updated every 5 min, allowed dose adjustments to be timed to the increasing limb of hyperglycemic excursions rather than to their peak. This form of anticipatory titration that reduces both overshoot and correction-related hypoglycemia; this system-level advantage is inaccessible with conventional intermittent point-of-care measurements.

Device insertion required adaptation: a subcutaneous fat depth of only 1–2 mm rendered standard perpendicular insertion inapplicable; near-horizontal tangential manual insertion achieved reliable positioning.

The 76%–88% reduction in cutaneous punctures represents a clinically meaningful reduction in procedural pain; in a patient of this size and fragility, each heel stick constitutes a significant nociceptive stressor with immediate hemodynamic and long-term neurodevelopmental implications (15).

The clinical complexity of this case—Candida septicemia, FIP, and recurrent pulmonary hypertension—demonstrates that sensor-augmented pump therapy can be sustained throughout critical illness.

A particularly noteworthy aspect is the uninterrupted continuation of both CGM and subcutaneous pump therapy throughout a 1-h 22-min laparotomy, which is, to our knowledge, the first documented instance in an ELBW neonate. Subcutaneous delivery is independent of the intravenous circuit, eliminating competition for critically limited venous access. CGM provides real-time glycemic surveillance and enables proactive insulin dose titration throughout the operative period, in contrast to conventional intermittent monitoring, which permits only reactive adjustments. No hypoglycemia occurred during the operative window.

The nursing staff confirmed that routine care was not impeded by the devices. The infusion set is certified for use for up to 7 days, substantially reducing the frequency of insulin preparation, line handling, material consumption, and contamination risk compared to the 12-hourly syringe changes required by intravenous protocols.

Despite continuous monitoring, glycemic control remained suboptimal, with a TIR of 59.4%, reflecting the inherent limitations of manual titration and indicating a substantial potential for improvement through algorithm-driven approaches. Notably, Böettger et al. reported normoglycemia in only 15.6% of a comparable CSII cohort managed without CGM (14), suggesting that real-time trend data are essential for effective subcutaneous insulin management.

Beardsall et al. demonstrated that a fully automated closed-loop system increased the time in the target range (72–144 mg/dL; 4–8 mmol/L) from 26% to 91% in extremely preterm infants weighing below 1,200 g (16). Although this system used a different target range and intravenous dextrose co-infusion model, sensor-driven automated insulin adjustment is directly transferable to subcutaneous CSII.

Combined CGM and CSII therapy is technically feasible in ELBW infants. Compared with intravenous insulin, sensor-augmented pump therapy provides continuous real-time glucose monitoring, trend-guided titration, and independence from dedicated vascular access, while establishing the anatomical and procedural foundation for purpose-built closed-loop algorithms in ELBW infants. Uninterrupted perioperative glycemic surveillance during laparotomy is feasible without additional invasive procedures. These findings highlight an unmet clinical need and support the development of neonatal-adapted CGM and CSII devices; prospective studies are warranted.

## Data Availability

The original contributions presented in this study are included in the article. Further inquiries can be directed to the corresponding author.

## References

[1] Ogilvy-Stuart AL Beardsall K. Management of hyperglycaemia in the preterm infant. Arch Dis Child Fetal Neonatal Ed. (2010) 95(2):F126–31. 10.1136/adc.2008.154716 20231218

[2] Alexandrou G Skiöld B Karlén J Tessma MK Norman M Ådén U. Early hyperglycemia is a risk factor for death and white matter reduction in preterm infants. Pediatrics. (2010) 125(3):e584–91. 10.1542/peds.2009-0449 20176674

[3] Hays SP Smith EO Sunehag AL. Hyperglycemia is a risk factor for early death and morbidity in extremely low birth-weight infants. Pediatrics. (2006) 118(5):1811–8. 10.1542/peds.2006-0628 17079549

[4] Beardsall K Vanhaesebrouck S Ogilvy-Stuart AL Vanhole C Palmer CR van Weissenbruch M. Early insulin therapy in very-low-birth-weight infants. N Engl J Med. (2008) 359(18):1873–84. 10.1056/NEJMoa0803725 18971490

[5] Bottino M Cowett RM Sinclair JC. Interventions for treatment of neonatal hyperglycemia in very low birth weight infants. Cochrane Database Syst Rev. (2011) 10:CD007453. 10.1002/14651858.CD007453.pub3 21975769

[6] Zahid N Taylor KM Gill H Maguire F Shulman R. Adsorption of insulin onto infusion sets used in adult intensive care unit and neonatal care settings. Diabetes Res Clin Pract. (2008) 80(1):e11–3. 10.1016/j.diabres.2008.02.013 18395926

[7] Knopp JL Bishop K Lerios T Chase JG. Capacity of infusion lines for insulin adsorption: effect of flow rate on total adsorption. J Diabetes Sci Technol. (2021) 15(1):124–31. 10.1177/1932296819876924 PMC7783010 31561709

[8] Hewson M Nawadra V Oliver J Odgers C Plummer J Simmer K. Insulin infusions in the neonatal unit: delivery variation due to adsorption. J Paediatr Child Health. (2000) 36(3):216–20. 10.1046/j.1440-1754.2000.00488.x 10849219

[9] Perri A Giordano L Corsello M Priolo F Vento G Zecca E. Continuous glucose monitoring in very low birth weight newborns needing parenteral nutrition: validation and glycemic percentiles. Ital J Pediatr. (2018) 44(1):146. 10.1186/s13052-018-0542-5 30134937 PMC6106728

[10] Galderisi A Facchinetti A Steil GM Ortiz-Rubio P Cavallin F Tamborlane WV. Continuous glucose monitoring in very preterm infants: a randomized controlled trial. Pediatrics. (2017) 140(4):e20171162. 10.1542/peds.2017-1162 28916591

[11] Beardsall K Thomson L Guy C Iglesias-Platas I van Weissenbruch MM Bond S. Real-time continuous glucose monitoring in preterm infants (REACT): an international, open-label, randomised controlled trial. Lancet Child Adolesc Health. (2021) 5(4):265–73. 10.1016/S2352-4642(20)30367-9 33577770 PMC7970623

[12] Desenfants A Soleirol M Salet R Benito Castro F Le Guillou C Di Maio M. Efficiency of continuous subcutaneous insulin infusion for premature neonate: a case report. Neonatology. (2022) 119:260–3. 10.1159/000521695 35130548

[13] Becocci A Bochaton N Fau S Klee P Perrenoud L Fonzo-Christe C. Continuous subcutaneous insulin infusion via an insulin pump in extremely premature neonates—a case series. Intensive Care Med Paediatr Neonatal. (2023) 1(3). 10.1007/s44253-023-00004-3

[14] Böettger M Zhou T Knopp J Chase JG Heep A von Vangerow M. Treatment of severe hyperglycemia in extremely preterm infants using continuous subcutaneous insulin therapy. J Clin Res Pediatr Endocrinol. (2024) 16(4):443–9. 10.4274/jcrpe.galenos.2024.2024-2-9 38915194 PMC11629728

[15] Grunau RE. Neonatal pain in very preterm infants: long-term effects on brain, neurodevelopment and pain reactivity. Rambam Maimonides Med J. (2013) 4(4):e0025. 10.5041/RMMJ.10132 24228168 PMC3820298

[16] Beardsall K Thomson L Elleri D Dunger DB Hovorka R. Feasibility of automated insulin delivery guided by continuous glucose monitoring in preterm infants. Arch Dis Child Fetal Neonatal Ed. (2020) 105:F279–84. 10.1136/archdischild-2019-316871 PMC7363782 31399480

